# Sociodemographic, clinical, and psychosocial factors associated with depression among type 2 diabetic outpatients in Black Lion General Specialized Hospital, Addis Ababa, Ethiopia: a cross-sectional study

**DOI:** 10.1186/s12888-016-0809-6

**Published:** 2016-04-15

**Authors:** Tesfa Dejenie Habtewold, Sisay Mulugeta Alemu, Yohannes Gebreegziabhere Haile

**Affiliations:** Department of Nursing, College of Health Science, Debre Berhan University, Debre Berhan, P.O. BOX: 445, Ethiopia; International Medical Corps, Mental Health and Psychosocial Support Program, Dollo Ado Refugee Camp, Ethiopia

**Keywords:** Diabetes, Depression, Sociodemographic factors, Clinical factors, Psychosocial factors, Outpatient, Ethiopia, Cross-sectional study

## Abstract

**Background:**

Depression is a common comorbidity among patients with type 2 diabetes. There are several reports supporting a bidirectional association between depression and type 2 diabetes. However, there is limited data from non-western countries. Therefore, the aim of this study was to assess the sociodemographic, clinical, and psychosocial factors associated with co-morbid depression among type 2 diabetic outpatients presenting to Black Lion General Specialized Hospital, Addis Ababa, Ethiopia.

**Methods:**

This institution based cross-sectional study design was conducted on a random sample of 276 type 2 diabetic outpatients. Type 2 diabetes patients were evaluated for depression by administering a validated nine-item Patient Health Questionnaire (PHQ-9). Risk factors for depression among type 2 diabetes patients were identified using multiple logistic regression analysis.

**Result:**

In total, 264 study participants were interviewed with a response rate of 95.6 %. The prevalence of depression was 44.7 %. In the multivariate analysis, the statistically significant risk factors for depression were monthly family income ≤ 650 (*p*-value = 0.056; OR = 2.0; 95 % CI = 1.01, 4.2), presence of ≥3 diabetic complications (*p*-value = 0.03; OR = 3.3; 95 % CI = 1.1, 10.0), diabetic nephropathy (*p*-value = 0.01; OR = 2.9; 95 % CI = 1.2, 6.7), negative life events (*p*-value = 0.01; OR = 2.4; 95 % CI = 1.2, 4.5), and poor social support (*p*-value = 0.001; OR = 2.7; 95 % CI = 1.5, 5.0).

**Conclusion:**

This study demonstrated that depression is a common co-morbid health problem with a prevalence rate of 44.7 %. The presence of diabetic complications, low monthly family income, diabetic nephropathy, negative life event, and poor social support were the statistically significant risk factors associated with depression. We presume that the burden of mental health especially depression is high in the population with type 2 diabetes mellitus co-morbidity. Therefore, specific attention is needed to diagnose early and treat promptly.

**Electronic supplementary material:**

The online version of this article (doi:10.1186/s12888-016-0809-6) contains supplementary material, which is available to authorized users.

## Background

The mental health consequences of diabetes have been studied by a number of research teams and they have reported bidirectional associations between depression and type 2 diabetes [1, 2]. In this study, we focused on the influence of type 2 diabetic associated problems on the presence of depressive symptoms. Based on the literature we classify these potential risk factors as sociodemographic, clinical, and psychosocial factors.

Several studies have shown that sociodemographic risk factors affect the development of co-morbid depression among diabetic patients. The prevalence rates of depression were significantly higher in females with type 2 diabetes mellitus compared with males with type 2 diabetes mellitus [3–6], varying from a doubled percentage for women than for men [7] and more than three times higher in women compared with men [3]. Other sociodemographic risk factors that were significantly associated with depression in people with diabetes includes age at diabetes diagnosis [8, 9], low socioeconomic status [3, 4, 6, 10], low educational status [1, 3, 4], being unmarried [1, 4, 9], urban dwelling [3], nature of relationship with sexual partners [11], ethnicity/race [12, 13], smoking habits [14–16], physical activity [14], sedentary life [8], and unemployment [17]. Some other studies, however, found that gender, age, residence, educational status, ethnicity, marital status, employment status, and socioeconomic status had no significant association with depression in diabetes patients [11, 17–19].

The effects of clinical and psychosocial factors on co-morbid depression have been supported by different studies as well. These factors encompass financial stress [1, 19, 20], poor social support [1, 4], negative life events [1, 19, 20], poor quality of life [20, 21], and medication burden [19, 22].

Depression was most strongly associated with functional impairment [1], large waist circumference [19], glycosylated hemoglobin level [1, 3], body mass index [7, 12], blood glucose level [3], diabetic retinopathy [23], diabetic neuropathy [1, 12, 19, 23], diabetic nephropathy [19, 23], peripheral vascular disease [19, 23], diabetic foot ulcer [19], coronary vascular disease [23], ischemic heart disease [16, 23], arteriosclerotic vascular disease [23], heart disorder [3], type of diabetic treatment [3, 24], and sexual dysfunction [23]. On the contrary depression was not significantly associated with poor body weight control, insulin treatment users, duration of diabetes, glycosylated hemoglobin (HbA1c), obesity, hypertensive disorder, retinopathy [3, 12, 17, 18].

The major barriers in diagnosing depression among type 2 diabetic patients were the lack of screening tool, time constraints, and overlapping of physical and cognitive symptoms [3, 25].

Thus, there are various sociodemographic, clinical, and psychosocial factors related to depression in type 2 diabetes mellitus. Identifying the significant predictors of depression is important to develop need-based clinical and community-based mental health interventions. Although these factors have been found to be associated with depression, most of these studies were based on Western samples, with only some exceptions. Therefore, we do not know whether these findings can be generalized to a non-western setting. Thus in the present study, we aimed to identify factors influencing the risk of co-morbid depression among patients with type 2 diabetes mellitus in patients treated at the Black Lion General Specialized Hospital in Ethiopia. We hypothesized that the same identified factors were associated with depression in this non-western sample.

## Methods

### Study site

Black Lion General Specialized Hospital (Tikur Anbessa in Amharic), located in the nation’s capital Addis Ababa, is Ethiopia’s largest general public hospital and one of University Hospitals in the country. The hospital provides a tertiary level referral treatment and is open for 24 h for emergency services. Black Lion hospital offers diagnosis and treatment for approximately 370,000- 400,000 patients a year [26].

### Subjects and procedures

We conducted an institution based cross-sectional study design among 276 type 2 diabetic outpatients selected using systematic random sampling techniques from April 23, 2013 to May 13, 2013. Inclusion criteria were: (i) diagnosed as type 2 diabetic patients for at least one year, (ii) age ≥ 20 years old, and (iii) capable of independent communication and giving informed verbal consent. Individuals who were currently being treated for depression or other psychological problems (e.g. anxiety or personality disorders) as ascertained at recruitment were excluded. Patients with established type 2 diabetes mellitus were evaluated for depression by administering a validated nine-item questionnaire PHQ-9 (Amharic version-local language) [29].

#### Variables

The outcome variable is depression. The explanatory variables are sociodemographic, clinical, and psychosocial factors that were collected by patient interview and medical record review.

#### Tool reliability assessment

Cronbach’s α for the PHQ-9 scale was 0.72 indicating acceptable consistency of this psychometric scale for the study population. The correlations between nine items of the PHQ-9 and total PHQ-9 scores ranged from 0.22 to 0.69, and all correlations were significant at the 0.01 level.

### Data analysis

After checking collected data visually for completeness, the response was coded and entered into the computer using EPI info version 3.5.1. Statistical packages, and then 10 % of the responses was randomly selected and checked for the consistency of data entry. Then printed frequencies were used for checking of outliers and to clean data. Data was cleaned accordingly and then exported to SPSS (Statistical package for Social Science) version 20.0 (IBM SPSS Corp.). Cleaned raw data is available as an additional file in a comma delimited (*.csv) format [Additional file 1]. The frequency distribution of dependent and independent variables was worked out. Multivariate logistic regression analysis was applied to identify statistically significant associated risk factors using significance level (α) 0.05. Odds ratios were calculated to determine the strength of associations of selected variables. The study was adherent to the STROBE criteria as outlined in Additional file 2.

## Result

### Sociodemographic characteristics

In total, 264 study participants were interviewed with a response rate of 95.6 %. Non response was due to participant’s lack of interest to participate, shortage of time, and critical illness. Of the interviewed participants 53.0 % (*n* = 140) were female, 69.3 % (*n* = 183) were married, 80.7 % (*n* = 213) were Orthodox Christian, and 57.2 % (*n* = 151) were Amhara. In addition, the mean ± SD age at diagnosis and current age of the subjects were 43.9 ± 10.9 and 55.9 ± 10.9 years, respectively. Besides, 86.4 % (*n* = 228) lived in Addis Ababa, and 61.7 % (*n* = 163) had a waist circumference of ≥ 95 cm (mean ± SD, 98.9 + 11.1). The median monthly income of the family was 750 ETB (651–1400 ETB) and 33.7 % (*n* = 89) had attended college/university level education.

#### Clinical characteristics

One hundred forty five patients (43.2 %) were on oral hypoglycemic treatment, 78.3 % (*n* = 141) had cardiovascular diseases (hypertension and heart failure), and 69.7 % (n = 140) had diabetic retinopathy. More than half (58.7 %, *n* = 155) of study participants reported 1 to 2 co-morbid disease (mean ± SD, 1.1 ± 0.9). Similarly, half of the respondents (*n* = 132) had a BMI ≤ 24.9 kg/m^2^ (mean ± SD, 25.4 ± 3.7). Regarding the laboratory reported fasting blood glucose level, 12.9 % (*n* = 34) had ≤ 100 mg/dl, 19.7 % (*n* = 52) 101–126 mg/dl, and 67.4 % (*n* = 178) had ≥ 127 mg/dl.

#### Prevalence of depression

The mean ± SD of PHQ 9 score was 5.2 ± 4.6. Twenty-five (9.5 %) type 2 diabetic outpatients did not report any of depressive symptoms, but 28.4 % of type 2 diabetic outpatients, fulfilled the criteria for mild depression, 12.1 % for moderate depression, 2.7 % for moderately severe depression, and 1.5 % for severe depression. Thus, the overall prevalence rate of depression was 44.7 %.

#### Statistical analysis

As revealed in Table 1, the prevalence of depressive symptoms was two times higher in female type 2 diabetic patients compared with male. This was found statistically significant. Other demographic variable that found risk for depression and statistically significant was monthly family income.

**Table 1:** Binary logistic regression examining the association between sociodemographic factors and depressive symptoms, June 2013

Variables	Depression status		Univariate	
	Depressed (PHQ 9 ≥ 5) n/%	Not depressed (PHQ 9 ≤ 4) n/%	*p*-Value	OR(95 % CI)
Sex, female	74/52.8	66/48.2	0.005	2.0(1.2, 3.4)
Current age, ≥65	30/50	30/50	0.59	1.2(0.7, 2.1)
Age at diagnosis, ≤44	68/49.6	69/50.4	0.30	1.3(0.8, 2.4)
Residence, outside Addis Ababa	16/44.4	20/55.6	0.97	1.0(0.5, 2.1)
Marital status				
Married	72/39.3	111/61.7	0.18	0.7(0.3, 1.2)
Divorced	16/66.7	8/33.3	0.18	2.0(0.7, 5.6)
Ethnicity, Amhara	67/44.4	84/55.6	0.50	0.7(0.3, 2.0)
Educational status				
Grade 1–8	41/63.1	24/36.9	0.16	1.8(0.8, 4.2)
Grade 9–12	21/35.6	38/64.4	0.22	0.6(0.3, 1.4)
Monthly income (ETB)***				
≤650	66/52.8	59/47.2	0.001	2.7(1.5, 5.0)
651–1400	29/47.5	32/52.5	0.03	2.2(1.1, 4.4)
Waist circumference ≥95 cm	80/49.1	83/50.9	0.07	1.6(1.0, 2.6)

*** = 1 US$, 18 Ethiopian birr

As shown in Table 2, among clinical variables the presence of diabetic complications (nephropathy, neuropathy, sexual dysfunction), body mass index 25.0–29.9 kg/m^2^ and, the presence of ≥3 co-morbid disease, and occurrence of 1–2 diabetic complication were found statistically significant risk factors of depression.

**Table 2:** Binary logistic regression examining the association between clinical factors and depressive symptoms, June 2013

Variables	Depression status		Univariate	
	Depressed (PHQ 9 ≥ 5) n/%	Not depressed (PHQ 9 ≤ 4) n/%	*p*-Value	OR(95 % CI)
Diabetes treatment				
Insulin plus oral hypoglycemic	15/50	15/50	0.93	1.0(0.5, 2.3)
Oral hypoglycemic	43/37.7	71/62.3	0.09	0.6(0.4, 1.1)
Cardiovascular disease	69/48.9	72/51.1	0.14	1.5(1.0, 2.4)
Other co-morbid disease	40/50	40/50	0.25	1.3(0.8, 2.3)
Diabetic retinopathy	69/49.3	71/50.7	0.11	1.5(0.9, 2.4)
Diabetic nephropathy	44/63.8	25/36.2	0.000	3.0(1.6, 5.1)
Diabetic neuropathy	45/54.2	38/45.8	0.03	1.8(1.0, 3.0)
Sexual dysfunction	22/31.9	47/68.1	0.01	0.5(0.3, 0.8)
Duration of diabetes, 9–16 years	46/47.9	50/52.1	0.37	1.3(0.7, 2.3)
Duration of diabetes treatment, 9–16 years	43/46.2	50/53.8	0.63	1.2(0.6, 2.0)
Fasting blood sugar ≥127 mg/dl	84/47.2	94/52.8	0.13	1.3(0.6, 2.7)
Body mass index (kg/m^2^ )				
25.0–29.9	50/51	48/49	0.02	1.8(1.1, 3.1)
≥30	20/58.8	14/41.2	0.02	2.5(1.2, 5.4)
Number of co-morbidity,				
1–2	66/42.6	89/57.4	0.81	1.1(0.6, 1.8)
≥3	18/69.2	8/30.8	0.01	3.2(1.3, 8.3)
Pill burden				
5–6 pills	47/41.6	66/58.4	0.85	1.1(0.6, 1.9)
≥7 pills	40/54.1	34/45.9	0.09	1.8(0.9, 3.3)
Number of diabetic complication				
1–2	72/48.7	76/51.3	0.04	2.0(1.0, 3.4)
≥3	27/50	27/50	0.15	1.7(0.8, 3.7)
Physical disability	63/47.7	69/52.3	0.32	1.3(0.8, 2.1)

Negative life events in the last 6 months and poor social support were psychosocial risk factors significantly associated with depression. But, not fearing diabetic complication and doing daily recommended physical exercise were statistically significant preventive factors of depression among type 2 diabetic patients. This was presented in Table 3 below.

**Table 3:** Binary logistic regression examining the association between psychosocial factors and depressive symptoms, June 2013

Variables	Depression status		Univariate	
	Depressed (PHQ 9 ≥ 5) n/%	Not depressed (PHQ 9 ≤ 4) n/%	*p*-Value	OR(95 % CI)
Negative life event	53/60.2	35/39.8	0.000	2.6(1.5, 4.4)
Poor social support	59/62.1	36/37.9	0.000	3.1(1.8, 5.2)
Fear of diabetic complication				
Yes	89/50	89/50	0.65	0.8(0.3, 2.1)
No	19/27.9	49/72.1	0.03	0.3(0.1, 0.9)
Health care cost				
High	85/44.3	107/55.7	0.18	0.5(0.2, 1.4)
Not high	22/40.7	32/59.3	0.14	0.4(0.2, 1.3)
Doing physical activity	17 (30.4 %)	39 (69.6 %)	0.02	0.5(0.3, 0.9)

Table 4 depicted that, only monthly family income, presence of ≥3 diabetic complication, diabetic nephropathy, negative life event, and poor social support persisted as statistically significant risk factors for depression after controlling of other confounding factors.

**Table 4:** Multivariate logistic regression examining the relation between different associated factors and depressive symptoms, June 2013

Variables	Depression status		Multivariate	
	Depressed (PHQ 9 ≥ 5) n/%	Not depressed (PHQ 9 ≤ 4) n/%	*p*-Value	OR(95 % CI)
Sex, female	74/52.8	66/48.2	0.33	0.7(0.4, 1.4)
Monthly income (ETB)***				
≤650	66/52.8	59/47.2	0.056	2.0(1.01, 4.2)
651–1400	29/47.5	32/52.5	0.26	1.6(0.7, 3.6)
Body mass index (kg/m^2^)				
25.0–29.9	50/51	48/49	0.32	0.6(0.2, 1.6)
≥30	20/58.8	14/41.2	0.71	1.2(0.5, 3.1)
Number of co-morbidity, ≥3	18/69.2	8/30.8	0.03	0.3(0.1, 0.9)
Number of diabetic complication, ≥3	27/50	27/50	0.03	3.3(1.1, 10.0)
Diabetic neuropathy	45/54.2	38/45.8	0.42	1.4(0.6, 3.2)
Diabetic nephropathy	44/63.8	25/36.2	0.01	2.9(1.2, 6.7)
Sexual dysfunction	22/31.9	47/68.1	0.60	1.3(0.5, 3.0)
Negative life event	53/60.2	35/39.8	0.01	2.4(1.3, 4.5)
Poor social support	59/62.1	36/37.9	0.001	2.7(1.5, 5.0)
Fear of diabetic complication	89/50	89/50	0.65	1.3(0.4, 4.1)
Doing physical activity	17/30.4	39/69.6	0.48	0.8(0.3, 1.7)

*** = 1 US$, 18 Ethiopian birr

## Discussion

In the general diabetic population, it is difficult to accurately estimate the potential medical care needs and public health burdens of depression [28]. Unfortunately, in spite of the high impact of depression and diabetes comorbidity on the individual and its importance as a public health problem, little is known about the existence of depression in people with diabetes in Ethiopia. To our knowledge, this is the first study to assess sociodemographic, clinical, and psychosocial factors related to type 2 diabetes mellitus causing depression in a large sample. This study has tried to address this issue by identifying factors associated with depression in type 2 diabetic outpatients.

In this study low family monthly income was the significant independent predictor of depression. This result was consistent with other studies report [3, 4, 6, 10].

We found that sex, residence, marital status, ethnicity, educational status, waist circumference, current age, and age at diagnosis were not associated factors for depression. This study finding was also in line with several previous studies [11, 14, 17, 27].

However, many earlier published articles reported the prevalence rates of depression were significantly higher in females with type 2 diabetes mellitus compared with males with type 2 diabetes mellitus [3–5, 8–11]. Other demographic risk factors that were significantly associated in varying degree with depression in people with diabetes includes age at diabetes diagnosis [8, 9], low educational level [1, 3, 4], being unmarried [1, 4, 7], urban residence [3], nature of relationship with sexual partners [11], ethnicity/race [12, 13], smoking habits [14–16], physical activity [14], sedentary life [8], and unemployment [17]. This discrepancy was might be due to variation in study design, demographic characteristics of respondents, and selection method of respondents.

Regarding the clinical characteristics of participants, earlier studies conducted in different setting revealed depression was most strongly associated with large waist circumference [19], body mass index [7, 12], blood sugar level [3], diabetic retinopathy [23], diabetic neuropathy [1, 12, 19, 23], diabetic nephropathy [19, 23], peripherovascular disease [19, 23], diabetic foot ulcer [19], coronary vascular disease [23], ischemic heart disease [16, 23], arteriosclerotic vascular disease [23], heart disorder [3], type of diabetic treatment [3, 24], and sexual dysfunction [23].

Similarly, the result of this study demonstrated a significantly higher prevalence of depression in type 2 diabetic outpatients with the presence of ≥3 diabetic complication and diabetic nephropathy.

On the contrary depression was not significantly associated with diabetes treatment regimen co-morbid disease (cardiovascular, respiratory, renal, neurologic), complication of diabetes (retinopathy, neuropathy, sexual dysfunction), duration of diabetes, duration of diabetes treatment, fasting blood sugar, body mass index, number of co-morbidity, number of prescribed medication administration per day, and physical disability. This variation might be due to the level of country development, time frame, study setting, and lifestyle variation.

Moreover, we found out negative life events and poor social support were a statistically significantly psychosocial risk factor for depression. In line with this study the psychosocial factors that had a significant association with co-morbid depression comprises poor social support [2, 4] and experience chronic stressors or negative life events [2, 14, 17].

Previous studies also support increased health care costs/financial stress [2, 14, 20] and pill burden [14, 24] were associated factors for depression. Disparately, in this study high health care cost and medication burden were not associated with depression.

The outcomes of this study have implications for health care practice in Black Lion General Specialized Hospital and other health care organizations, where clinician’s diagnosis of psychiatric disorders rate is inadequate because of the high patient load, lack of screening tool, and poor undergraduate or in-service training in these skills. Principally this study has a presumption that the burden of mental health especially depression is high in the population with type 2 diabetes mellitus co-morbidity and requires attention to diagnose early and treat promptly. Laboratory test results and pharmacological treatment plan are not adequate to scaling up service delivery and bring about the expected change.

### Limitations and strengths

The strengths of this study include a high response rate and the inclusive nature of this research as individuals could participate regardless of literacy level. Including patients from different ethnic backgrounds in Addis Ababa and outside Addis Ababa was a further strength. Additionally, rather than having to rely on self-report, health-related information was collected from patients’ medical records. Even though the association was temporary, depression and type 2 diabetes mellitus were causally related and deserves attention from clinicians to ensure better management. Also, a reasonable sample size and ascertaining depression with culturally standardized questionnaires are strengths of this study. Since it was the first study in type, it will provide basic information for those who are interested.

However, an important limitation of this study was that a psychiatric diagnostic interview which is considered as the gold standard for the diagnosis of depression was not used. Additionally, there was an absence of similar study done in Ethiopia health care setting to compare the finding. Moreover, due to cross-sectional nature of the study, causal relationships between depression and type 2 diabetes mellitus cannot be assumed.

## Conclusion

In conclusion, this study demonstrated that depression is a common co-morbid health problem in type 2 diabetic out-patients in Black Lion General Specialized Hospital with a prevalence rate of 44.7 %. Within this sample of outpatients with type 2 diabetes mellitus, the study found that low monthly family income, presence of ≥3 diabetic complications, diabetic nephropathy, negative life event, and poor social support were highly statistically significant risk factors associated with depression. All the result should be interpreted cautiously and further prospective longitudinal research focusses on these sociodemographic and clinical factors in the different clinical group should be conducted. Finally, we presumed the burden of mental health especially depression is high in the population with type 2 diabetes mellitus co-morbidity and requires attention to diagnose early and treat promptly.

### Ethics approval and consent to participate

In order to follow the ethical and legal standards of the scientific investigation, the study was conducted after the approval of the proposal by Addis Ababa University Institutional Review Board. Participation was voluntary and information was collected anonymously after obtaining written consent from each respondent by assuring confidentiality throughout the data collection period.

### Consent for publication

Not applicable.

### Availability of data and materials

The data is available to the concerned body when it is required.

## Additional files

Additional file 1: This is a cleaned comma delimited (*.csv) dataset titled “Patients sociodemographic, clinical, psychosocial, and depressive symptom raw data”. It comprises patients’ sociodemographic, clinical, psychosocial, and depressive symptoms information. All data was anonymized. (CSV 87 kb) Additional file 2: STROBE Statement. (DOC 89 kb)
